# The dynamic broad epigenetic (H3K4me3, H3K27ac) domain as a mark of essential genes

**DOI:** 10.1186/s13148-021-01126-1

**Published:** 2021-07-08

**Authors:** Tasnim H. Beacon, Geneviève P. Delcuve, Camila López, Gino Nardocci, Igor Kovalchuk, Andre J. van Wijnen, James R. Davie

**Affiliations:** 1 CancerCare Manitoba Research Institute, CancerCare Manitoba, Winnipeg, MB R3E 0V9 Canada; 2 Department of Biochemistry and Medical Genetics, University of Manitoba, 745 Bannatyne Avenue, Room 333A, Winnipeg, MB Canada; 3 Faculty of Medicine, Universidad de Los Andes, Santiago, Chile; 4 Molecular Biology and Bioinformatics Lab, Program in Molecular Biology and Bioinformatics, Center for Biomedical Research and Innovation (CIIB), Universidad de Los Andes, Santiago, Chile; 5 Department of Biological Sciences, University of Lethbridge, Lethbridge, AB Canada; 6 Department of Orthopedic Surgery, Mayo Clinic, Rochester, MN USA; 7 Department of Biochemistry and Molecular Biology, Mayo Clinic, Rochester, MN USA

**Keywords:** Broad H3K4me3 domains, Enhancers, Histone modifications, Epigenetics, Cell identity genes, Tumor suppressor genes

## Abstract

Transcriptionally active chromatin is marked by tri-methylation of histone H3 at lysine 4 (H3K4me3) located after first exons and around transcription start sites. This epigenetic mark is typically restricted to narrow regions at the 5`end of the gene body, though a small subset of genes have a broad H3K4me3 domain which extensively covers the coding region. Although most studies focus on the H3K4me3 mark, the broad H3K4me3 domain is associated with a plethora of histone modifications (e.g., H3 acetylated at K27) and is therein termed broad epigenetic domain. Genes marked with the broad epigenetic domain are involved in cell identity and essential cell functions and have clinical potential as biomarkers for patient stratification. Reducing expression of genes with the broad epigenetic domain may increase the metastatic potential of cancer cells. Enhancers and super-enhancers interact with the broad epigenetic domain marked genes forming a hub of interactions involving nucleosome-depleted regions. Together, the regulatory elements coalesce with transcription factors, chromatin modifying/remodeling enzymes, coactivators, and the Mediator and/or Integrator complex into a transcription factory which may be analogous to a liquid–liquid phase-separated condensate. The broad epigenetic domain has a dynamic chromatin structure which supports frequent transcription bursts. In this review, we present the current knowledge of broad epigenetic domains.

## Introduction

**Fig. 1:**
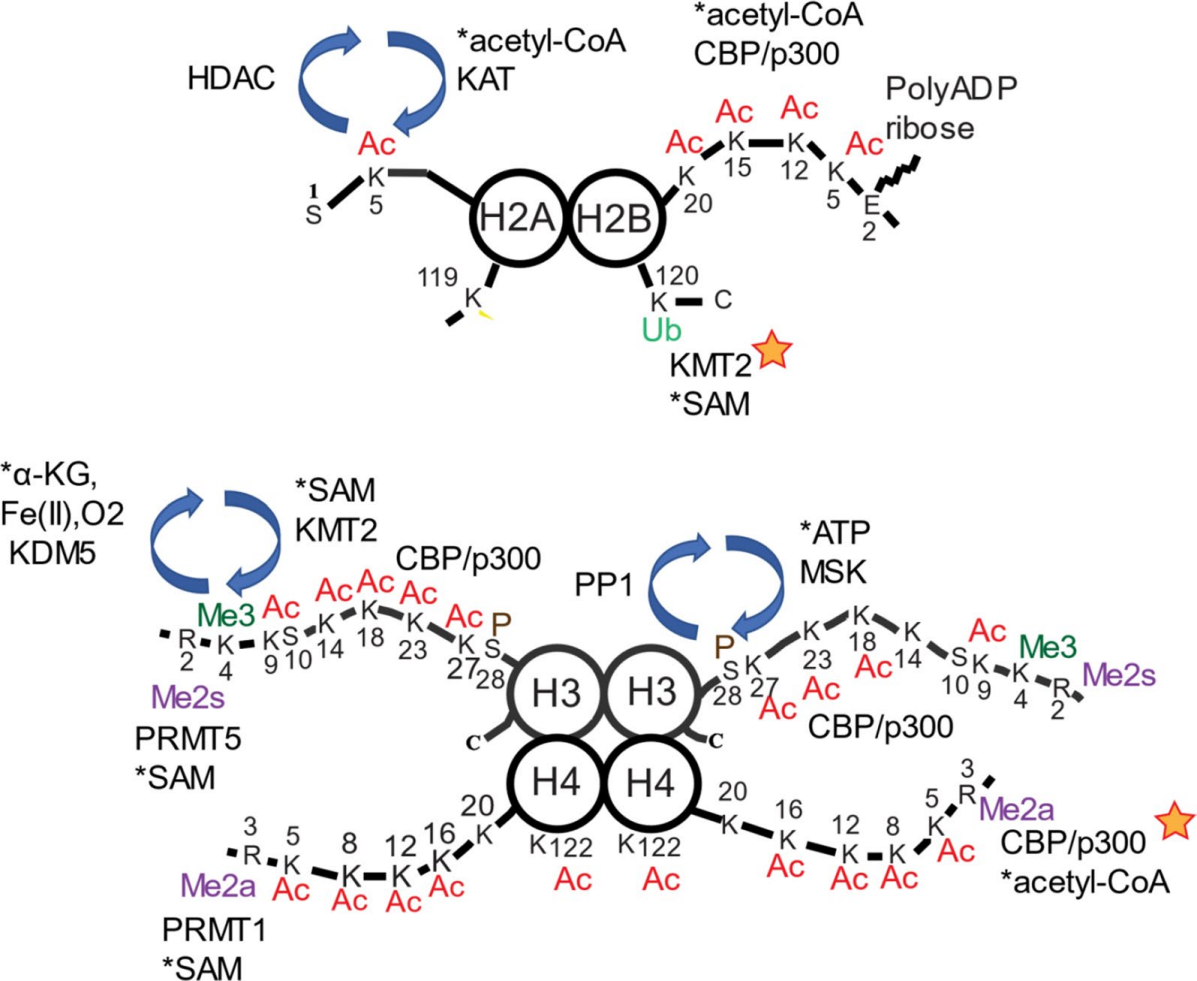
Histone post-translational modifications. The four core histones are modified by acetylation (Ac), phosphorylation (P), lysine trimethylation (Me3), arginine symmetric and asymmetric dimethylation (Me2s, Me2a, respectively), ubiquitination (Ub), and poly ADP ribosylation. The chromatin modifying enzymes shown are lysine acetyltransferases (KAT), histone deacetylases (HDAC), protein arginine methyltransferases (PRMT), lysine methyltransferases (KMT), mitogen- and stress-activated protein kinases (MSK), and protein phosphatase (PP). The metabolites (α-ketoglutarate, α-KG; S-adenosylmethionine, SAM) required for enzyme activity are indicated with an asterisk. The chromatin enzymes, which have elevated activity when binding a histone modification, are indicated with a star

Aside from being the structural unit in chromatin, the nucleosome is a signaling module responding to changes in metabolism and environmental conditions [1]. The nucleosomal histones, consisting of two each of the histones H2A, H2B, H3, H4, are susceptible to numerous post-translational modifications (PTMs) throughout their entire length (Fig. 1). The modifications either enhance or retard the binding of proteins which alter the structure and function of the chromatin region. Histone PTMs are key components of the critical epigenetic machinery that provides protein-based regulatory information beyond the regulatory cues embedded within the DNA sequence. Among the players regulating the epigenetic machinery are writers, readers, and erasers (http://www.epigeneticmachinery.org/) [2]. Writers are chromatin modifying enzymes that add the modification to histones, non-histone proteins, DNA, and RNA. Readers are proteins that read the cues written by the writer, while erasers represent enzymes that remove the chemical information embodied in the PTM. In the same way, metabolism and epigenetic processes are closely linked, for example, S-adenosyl-L-methionine (SAM) is required for lysine and arginine methyltransferases, acetyl-CoA for lysine acetyltransferases, iron and α-ketoglutarate for lysine demethylases, and ATP for histone kinases (for review see [3, 4]) (Fig. 1).

In the context of nucleosome signaling, histone H3 trimethylated at lysine 4 (H3K4me3) integrates a variety of signaling pathways, including transcription initiation, elongation, and RNA splicing [5]. H3K4me3, a modification that is associated with transcriptionally active/poised chromatin (referred to as an active histone mark), exhibits two distinct distribution patterns, both conserved in yeast, plants, worms, flies, and mammals [6–8]. In chromatin immunoprecipitation sequencing (ChIP-seq) experiments, most H3K4me3-enriched nucleosomes are detected as sharp, narrow peaks (< 1 kb) positioned near the transcription start sites (TSSs). Alternatively, a small subset of genes is associated with broad H3K4me3 domains (> 4 kb) extending downstream into their body and exhibiting a lower signal intensity than sharp H3K4me3 peaks. It was suggested that different mechanisms regulate the two H3K4me3 distribution patterns as sharp and broad H3K4me3 peaks do not overlap [9], for example, the binding of different transcription factors, of different chromatin remodelers, or a differential role for super-enhancers [10]. The nucleosomes of the broad H3K4me3 domain have many different histone PTMs [9]. Henceforth, this chromatin feature of expressed genes will be referred to as the broad epigenetic domain.

In this review, we will focus on the broad epigenetic domains in comparison with the known features of the prevalent H3K4me3 mark enriched at the 5’ end of active genes. We will first briefly summarize the known features of the prevalent H3K4me3 mark enriched at the 5’ end of active genes as well as the enzymes that catalyze deposition/removal (writers/erasers) of H3K4me3 and proteins (readers) that bind to H3K4me3. While these interactions have been mostly studied in the context of the sharp, narrow H3K4me3 peaks near the TSSs, this current understanding of sharp H3K4me3 domains provides a solid foundation for modeling mechanistic concepts related to regulatory proteins for the broad epigenetic domains.

## Function of H3K4me3 at the 5’end of active genes

H3K4me3-marked nucleosomes are detected as sharp, narrow peaks flanking the TSSs with their intensity correlating with transcriptional activity [11]. However, the predominant peak maps to the 5′ end of the body of active genes, in association with unmethylated CpG islands [5, 12, 13]. In mammalian cells, this predominant H3K4me3 peak is located at the end of the first exon at the site of the 5’ splice site [14]. The correlation between H3K4me3 peak intensities and transcription levels has been repeatedly demonstrated across species. Yet, there is a long-standing and fundamentally unresolved question about the functional significance of H3K4me3 and several mechanistic possibilities are plausible. H3K4me3 may be causative and instruct RNA polymerase II-mediated transcription or it could be the direct consequence of transcription. Alternatively, H3K4me3 deposition could be an independent concurrent event. Adding to this molecular intricacy, the role that H3K4me3 has on gene transcription may be context dependent [15]. The elegant studies of Cano-Rodriguez et al. [16] using epigenomic editing showed local induction of H3K4me3 resulted in expression of silenced genes. The authors used the SET domain of the Zebrafish histone methyltransferase PRDM9 fused to catalytically dead Cas9 or zinc finger proteins, which was directed to specific silenced promoters. As long as the targeted promoter was not hypermethylated, directing the methyltransferase and H3K4me3 was sufficient to activate gene expression. This work supports the view that H3K4me3 facilitates transcription initiation through the binding of the H3K4me3 reader TAF3, which is a subunit of TFIID, and the recruitment of the preinitiation complex.

In addition to RNA polymerase II-mediated transcription, H3K4me3 has been linked to pre-mRNA splicing, DNA recombination, and DNA repair [6, 7]. Hence, H3K4me3 may represent a conserved versatile epigenetic mark that could support multiple DNA or RNA related cellular processes.

## H3K4me3 writers, erasers, and readers

Although H3K4me3 is relatively stable and sustained potentially for hours and days, it is also a dynamic mark with its levels reflecting the balance between deposition by lysine methyltransferases (KMTs) and removal by lysine demethylases (KDMs) (Fig. 1). In mammals, there are three pairs of H3K4 methyltransferases homologous to yeast Set1: KMT2A/KMT2B, KMT2C/KMT2D, and KMT2F/G (a.k.a SETD1A and SETD1B, respectively). Each KMT2 isoform is present in a complex of auxiliary proteins required for activity and triggering the state (mono-, di-, or tri-) of methylation deposition. KMT2A-D are also known as MLL1-4. The current gene symbols for the MLL-related K4 methyl transferases are, respectively, KMT2D (MLL2), KMT2B (MLL4), KMT2C (MLL3), and KMT2A (MLL1). However, there is a confusion with the nomenclature between MLL2 and MLL4 [7, 17]. The current literature indicates that KMT2F and KMT2G complexes catalyze all three states of methylation and are responsible for most of H3K4 trimethylation including that at the broad H3K4me3 domains, while KMT2A-D produces mono- and/or di-methyl H3K4 [17–22] (Table 1). In one of these studies, H3K4 methyl forms were determined by mass spectrometry [19], circumventing the issue of frequent cross-reactivity of many H3K4me3 antibodies with H3K4me1 or H3K4me2 [23]. Hence, sophisticated biochemical findings support the notion that there are functional distinctions in the enzymatic activities of the KMT2A/B/C/D class versus the KMT2F/G class that may reflect potential distinctions in functions that may not yet be fully appreciated.

**Table 1:** Writers, erasers, and potential readers for broad H3K4me3 domains

Writers	Erasers	Readers
KMT2F (uH2B requirement)	KDM5A-D	Transcription regulation [TAF3, 4, 9 (TFIID complex), transcription factor (BPTF)-associated protein of 18 kDa (BAP18), Spindlin1, ING proteins]
KMT2G (no uH2B requirement)		Chromatin modification [PHF23 which recruits SIN3-HDAC, KDM5B; ING proteins which are associated with KATs and HDAC complexes, KATs (KAT3B, KAT2B, KAT7, KAT2A in SAGA complex), SGF29 (SAGA), KMTs (WDR5)]
		Chromatin remodeling (CHD1, NURF)
		Elongation (PAF, FACT)
		RNA splicing (U2 snRNP, U2AF65)
		Bromodomain proteins (BPTF)
		Protein members of Integrator and Mediator complexes
		Transcriptional processes (ADAR1)

**Fig. 2:**
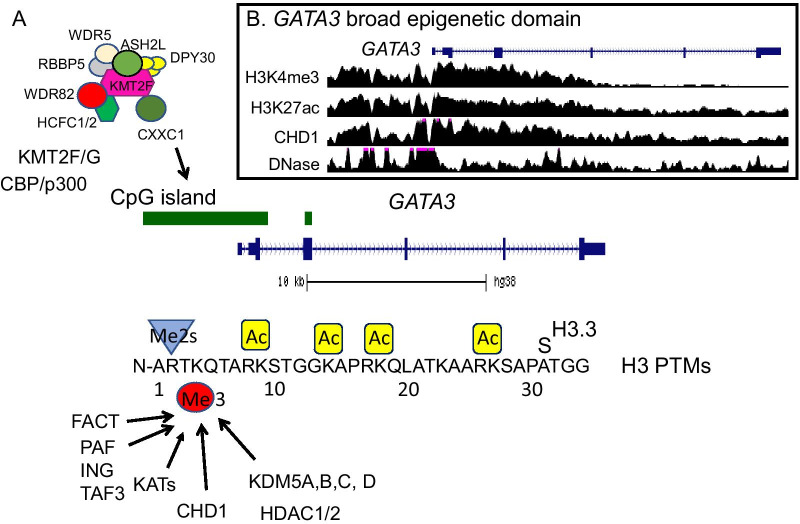
Histone H3K4me3 interactome. **A** KMT2F/G (SETD1A/B) are recruited via CXXC1 (CFP1) subunit to CpG islands. CBP/p300, which acetylates H3K27, binds to KMT2F/G. The H3K4me3-modified nucleosomes bind to multiple readers (CHD1, TAF3, ING, U2AF65, U2 snRNP, PAF, FACT, and KATs). KDM5 family members (erasers) are associated with HDAC1 and 2. The sequence of the first 34 amino acids of H3.3 is identical to the other H3s, except at amino acid position 31. **B** The broad epigenetic domain of the *GATA3* gene in MCF7 breast cancer cells. ChIP-Seq (H3K4me3, H3K27ac, CHD1) and DNase-seq tracks at the *GATA3* gene are shown [80]. Me2s, symmetric dimethylation, Ac, acetylation

The KMT2s are in large multiprotein complexes [21]. Core subunits of the KMT2 complexes are WDR5, ASH2L, RBBP5, and DPY30. The KMT2A/B complexes also have menin and HCFC1 or HCFC2; KMT2C/D complexes have PTIP, PA1, NCOA6 and UTX. The KMT2F/G complexes also consist of WDR82, HCFC1/2, and CXXC1 (a.k.a. CFP1) (Fig. 2A). BOD1L is present in the KMT2G, but not in the KMT2F complex [24]. Except for HCFC1/2, which do not appear in yeast, the proteins in these complexes are highly conserved between yeast, worms, plants, flies, and humans [6]. This extensive phylogenetic conservation indicates that the KMT2s have essential roles in the basic transcriptional machinery of any eukaryotic species, and KMT2 isoform diversification during evolution may parallel increased specialization in different cell types and tissues.

The transcription factors, basal transcription factors, modified histones, and long non-coding RNA interacting with the KMT2 subunits recruit these complexes to chromatin [21]. The CXXC1 subunit of the KMT2F/G complexes directs the enzyme to CpG islands and plays a key role in regulating gene expression through the formation of the H3K4me3 mark [25] (Fig. 2A). However, CXXC1 is also associated with expressed TSSs that lack CpG islands [26]. CXXC1 is a reader of the H3K4me3 mark, binding to this modified site via its PHD domain [15]. The absence of CXXC1 results in reduced transcription of CXXC1-occupied genes [15] and in disruption of cell differentiation (e.g., CD4+ T and T_H_17 cell differentiation) [25, 27]. In contrast to the maintenance of the H3K4me3 mark, establishment of this mark at highly transcribed genes may be through KMT2F/G complexes binding to the transcriptional machinery [15, 21].

Ubiquitinated H2B (uH2B) stimulates the activity of most KMT2s [28] (Fig. 1). Only KMT2G does not require uH2B to form H3K4me3 in an in vitro reconstituted chromatin system [28] (Table 1). Our group has previously shown that the ubiquitination of H2B is dependent on transcription [29]. Thus, transcription elongation, which supports the formation of uH2B, may be a prerequisite for the H3K4 trimethylation uH2B-dependent activity of KMT2F. Recently, it was reported that proteasome inhibition would result in the spreading of H3K4me3 into transcribed gene bodies [30]. Although the authors did not identify the specific epigenetic and transcriptional regulator(s) targeted by the proteasome, we offer the following for consideration. As proteasome inhibitors (e.g., MG132) result in the rapid deubiquitination of nucleosomal histones [31], KMT2G complexes may be responsible for the spreading of H3K4me3 as this H3K4 methyltransferase does not require uH2B for activity.

The molecular readers of the H3K4me3 mark bring a range of activities to modified chromatin regions [5]. The readers include protein/complexes that are involved in: (1) transcription regulation [TAF3 (TFIID complex), transcription factor (BPTF) associated protein of 18 kDa (BAP18), Spindlin1, ING proteins], (2) chromatin modification (PHF23 which recruits SIN3-HDAC, KDM5B; and ING proteins which are associated with KATs and HDAC complexes); (3) chromatin remodeling [chromodomain helicase DNA binding protein 1 (CHD1)]; (4) elongation [Polymerase-Associated Factor (PAF), Facilitates Chromatin Transcription (FACT)]; and (5) RNA splicing (U2 snRNP, U2AF65) [5, 32–37] (Fig. 2A) (Table 1). Several lysine acetyltransferases (KATs) (e.g., KAT2B, KAT7, KAT2A in SAGA complex) bind to H3K4me3 [5]. The recruitment of KATs to H3K4me3 nucleosomes results in heightened acetylation which is rapidly deacetylated by histone deacetylases (HDACs) (Fig. 2A). Inhibition of HDACs results in rapid hyperacetylation of the H3K4me3-modified nucleosome [38]. The KATs that may be involved in the hyperacetylation of H3K4me3-nucleosomes include those that bind to the H3K4me3 (KAT2B) and those that are associated with the KMT2s (CBP/p300) [38–41].

In humans, there are six H3K4 demethylases. KDM1A and B remove methyl groups from H3K4me1 and H3K4me2, but not from H3K4me3, while KDM5A-D (a.k.a. JARID1A-D) is able to remove methyl groups from each of the 3 states of H3K4 methylation [5, 7].

## Breadth of the broad epigenetic domain as a key chromatin signature

**Fig. 3:**
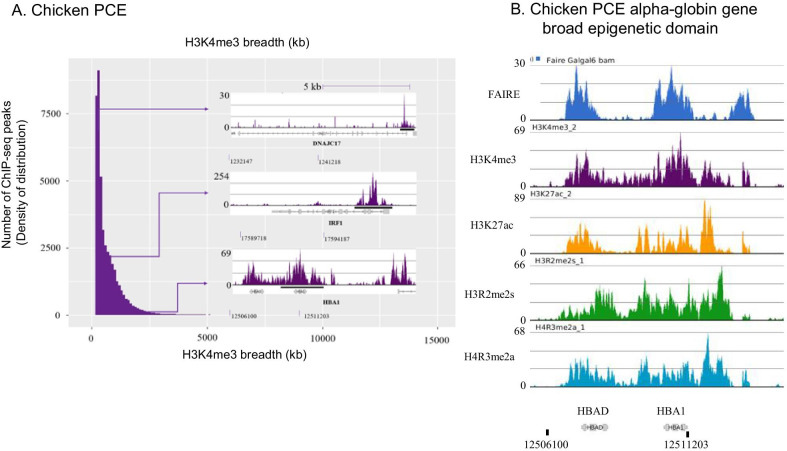
Multiple histone PTMs are associated with the broad epigenetic domain. **A** Breadth distributions of H3K4me3 ChIP-seq peaks in chicken polychromatic erythrocytes (PCE) [77]. **B** Chromatin profile of the *HBA1* (α-globin) genomic region. Partek chromosome view snapshot detailing the position of the FAIRE-seq peaks and H3K4me3, H3K27ac, H4R3me2a, and H3R2me2s ChIP-seq peaks. Transcripts (with exons as boxes) are depicted [47, 77]

In 2010, 2014 and 2015, the functional role of broad epigenetic domains with a focus on H3K4me3 was evidenced by statistical analyses of pooled high-throughput genomics data [9, 42–45]. A meta-analysis of H3K4me3 ChIP-seq data across > 20 different cell and tissue types in mice and humans, revealed a subset of genes marked by a broad H3K4me3 domain spanning the TSS. In identifying genes with the broad H3K4me3 domain, typically ChIP-seq peaks are called using the broad-peak function in MACS2 [9, 42–44]. The genes in the top 5% broadest H3K4me3 domains are then selected for further analyses as these broadest H3K4me3 domains discriminated cells or tissues according to their lineage in mammalian cells [42, 43] (Fig. 3A). These very broad H3K4me3 peaks were low signal intensity compared to narrow peaks [9]. The top 5% broadest H3K4me3 domains were enriched in genes involved in cell type-specific functions and cell identity, that is genes coding for factors essential to the establishment of cell lineage (see Glossary for definition of cell identity genes). This chromatin signature is conserved across species and taxa, from yeast to mammals including plants, flies, worms, and chicken [42, 43, 45, 46]. For examples, in human estrogen receptor positive (ER +) breast cancer MCF7 cells, the *GATA3* gene has a broad epigenetic domain (Fig. 2B). In chicken erythroid cells, genes [e.g., *IRF7*, *FTH1*, *HBBA* (β-globin), *HBA1* (α-globin)] have the broad epigenetic domain [47] (Fig. 3). Of note, the angiotensin-converting enzyme 2 (*ACE2*) gene, which codes for the receptor for the three coronaviruses HCoV-NL63, SARS-CoV and SARS-CoV-2, has a broad epigenetic domain in heart and small intestine, which are tissues with the highest *ACE2* expression [48].

Genes with the broad epigenetic domain displayed both enhanced transcriptional consistency and output. Single-cell RNA-seq showed that the variance in expression of genes with the top 5% broadest H3K4me3 domains was reduced compared to genes without the broad H3K4me3 domain [43]. Transcription is not a continuous process but occurs in bursts. Genes that are transcribed at higher rates have a greater number of transcriptional bursts. Among the burst parameters are burst fraction (the number of alleles in a cell that are being transcribed) and burst size (the number of transcripts generated per burst) [49]. The burst fraction relates to the burst frequency, i.e., how frequent bursts occur, since burst size and frequency are often the measures characterizing transcription bursting. Super-enhancers, which are required to maintain broad epigenetic domains, would increase both the burst size and fraction of the target gene [10, 49].

## Chromatin dynamics of broad epigenetic domains

The broad epigenetic domain has numerous histone PTMs, including H3K4me3, H3K23ac, H3K27ac, H3K79me1/2/3, H4K12ac, H4K20me1, H2BK5me, and H4R3me2a [9, 47, 50–52]. In addition to these histone PTMs, some broad epigenetic domains (e.g., *GATA3* and *FOXA1* in MCF7 cells) are associated with mitogen- and stress-activated protein kinase-generated H3S10ph and H3S28ph [53, 54]. The mitogen- and stress-activated protein kinase cooperates with CBP/p300 (gene symbol *EP300*) to produce H3K27ac/S28ph [55].

The dynamic histone modification of the broad epigenetic domain depends on the net activities of KATs/HDACs for acetylated histones, and KMTs/KDMs for methylated histones. The breadth of the broad H3K4me3 domain is dependent upon the net activity of KMT2F/G and KDM5A-D enzymes (Fig. 2A). Changes in the balance of these enzymes alter the breadth of the broad H3K4me3 domain and gene expression. For example, KDM5 inhibition results in increasing the broadness of promoter H3K4me3 peaks and increasing number of ER + breast cancer cells expressing the gene with the broad domain [52].

**Fig. 4:**
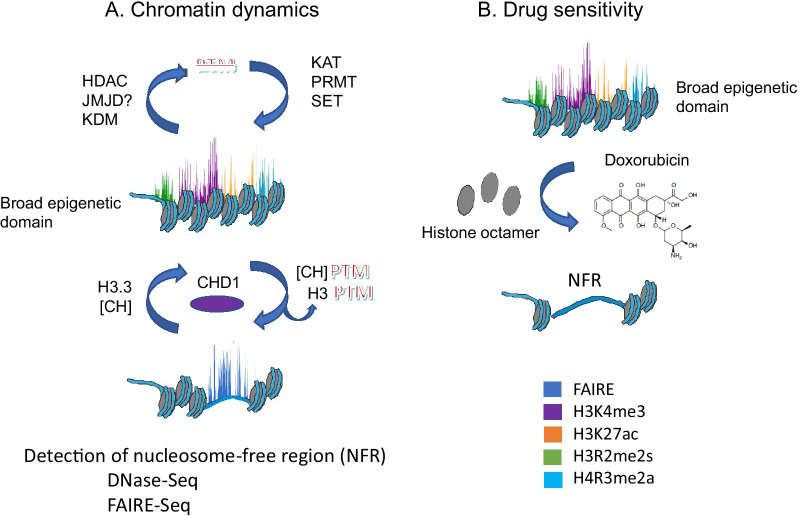
Model for nucleosome instability over the body of broad epigenetic domain genes. **A** Nucleosomes are being removed and reassembled due to the action of CHD1. Core histone [CH] PTMs would be removed with the displaced nucleosome (lower part of **A**) or by chromatin modifying enzymes (upper part of **A**). Jumonji domain containing 6 (JMJD6) is thought to demethylate H4R3me2a [136]. Nucleosome reassembly would allow for incorporation of histone variants such as H3.3 and a new set of core histone PTMs. Methods such as FAIRE-seq and DNase-seq will identify the nucleosome free regions. Regions lacking nucleosomes are detected by FAIRE-seq, and modified nucleosomes by ChIP-seq (color coded as in Fig. 3 to represent active marks present on all nucleosomes). The histone marks typically overlap over multi-modified nucleosomes but demonstrated individually for simplicity. **B** Doxorubicin intercalates into DNA of broad epigenetic domain displacing core histones. H3K4me3-modified nucleosomes are selectively dissociated when doxorubicin intercalates into these regions. This results in the formation of nucleosome-free regions (NFR) following the displacement of H3K4me3-modified nucleosomes

Considering the plethora of histone PTMs and the presence of chromatin remodelers (CHD1) [50], the broad epigenetic domain has an exceptionally dynamic chromatin structure (Fig. 4A), which would enable high transcription outputs and rapid response of inducible genes. The H3K4me3 modified nucleosomes are dynamically acetylated [38]. In the highly acetylated state, the H3K4me3 nucleosomes located downstream of the transcription start site would maintain an atypical nucleosome structure (called the U-shaped nucleosome) following the passage of RNA polymerase II [56, 57]. Such atypical nucleosome structures could support frequent rounds of transcription bursts.

CBP/p300 mediates the acetylation of the H3K4me3 nucleosome [38]. CBP/p300 acetylates multiple sites on the nucleosomal histones and regulates the acetylation of H2B (Ks on N-terminal tail) and H3 (K18, K27, K36) [58] (Fig. 1). Among the four core histones, H2B is the most dynamically acetylated [59]. In addition to the core histones, CBP/p300 acetylates numerous other proteins (referred to as acetyl-spray), several of which are involved in chromatin modification and transcriptional regulation [58]. Functional consequences of these acetylation events on the recipient proteins almost certainly occur and will require a major experimental effort to be fully deciphered. CBP/p300 co-condenses with transcription factors and could have a role in the transcriptional bursting of genes with the broad epigenetic domain [60].

The broad epigenetic domains have a nucleosome-free character as evidenced by FAIRE-seq and OCEAN-C [61, 62]. This feature of a destabilized chromatin structure for genes with the broad domain, which can be detected by FAIRE-seq or DNase-seq, is observed in vertebrate cells and is likely due to the action of CHD1 [46]. Figure 2B shows the association of CHD1 with the MCF7 *GATA3* broad epigenetic domain and the chromatin accessibility along the gene body as assayed through DNase-seq. CHD1, an ATP-dependent chromatin remodeler, is a reader of H3K4me3 and binds to the broad epigenetic domain [46, 50]. CHD1 is involved in the nucleosome turnover downstream of the transcription start site, regulates chromatin accessibility, and transcription elongation [63]. The remodeling activity of CHD1 promotes passage of RNA polymerase II through nucleosomes, alleviates RNA polymerase II pausing, facilitates high transcriptional outputs, and incorporation of the replacement histone H3.3 [64–67] (Fig. 4A).

CHD1 interacts with the H3K4me3 readers PAF, Mediator, FACT, and the U2 snRNP splicing complex. CHD1 remodeling activity is required to maintain uH2B levels and is required for the expression of differentiation-activated genes [66]. In maintaining the levels of uH2B, CHD1 activity would enhance the activities of the KMT2s (e.g., SETD1A) and perhaps plays a key role in maintaining the broad epigenetic domain [28].

The activity of CHD1 would result in nucleosome dissolution and reassembly, which could introduce the replacement histones such as H3.3 into the reassembled nucleosome as well as remove modified histones (Fig. 4A). H3.3 is named a replacement histone because it is expressed throughout most of the cell cycle and is available in the histone pools to form nucleosomes outside of S phase of the cell cycle. In contrast, most human histone genes are replication-dependent and are housed in large gene arrays on chromosomes 1 (13 genes) and 6 (55 genes). H3.3 is encoded by *H3F3A* and *H3F3B* genes that are not in the histone gene cluster. The H3.3 containing nucleosome would gain a richness of PTMs, consistent with previous reports [68].

Removal of the nucleosome via CHD1 also results in the removal of histone PTMs associated with the nucleosome, some of which are not readily de-modified (e.g., H4R3me2a) (Fig. 4A). The reassembly of the nucleosomes allows the chromatin modifying enzymes to lay down fresh histone PTMs to the newly formed histone octamer, which will contain the replacement histone variants, and the recruitment of readers of the histone PTMs.

With only two copies of the gene coding for H3.3, any mutation in these gene that impacts the modifications of this histone variant can have deleterious impacts on the genome, leading to cancer. Thus, these mutated histones are referred to as oncohistones [69]. The incorporation of mutant H3.3 (an oncohistone) in which the altered amino acids change PTMs could impact chromatin properties of the broad epigenetic domain and alter expression of genes with the broad epigenetic domain, contributing to diseases associated with H3.3 mutants [69, 70].

## Broad epigenetic domains and anticancer drugs

The destabilized chromatin structure of the broad epigenetic domain would make the broad epigenetic domain particularly vulnerable to the action of anticancer DNA intercalating drugs (Fig. 4B). Our studies reported that in vitro, the atypical nucleosomes associated with transcribed avian erythroid chromatin were sensitive to ethidium bromide dissociation [71]. In situ, H3K4me3-modified nucleosomes near promoters of transcribed genes were sensitive to dissociation by the anthracycline doxorubicin, a commonly used anticancer drug [72, 73]. The nucleosome dissociation and loss of H3K4me3 from human melanoma MelJuSo cells was observed after treatment with 9 uM doxorubicin for four hours. Further this selective dissociation of H3K4me3-modified nucleosomes and the increase in nucleosome-free regions were detected by an increase in FAIRE-seq peaks near promoter regions of active genes in MelJuSo cells [72]. The doxorubicin-mediated dissociation was independent of ATP and transcription [74]. In the administration of doxorubicin to breast cancer patients, cardiotoxicity is a major side effect [74]. The anticancer and cardiotoxicity of doxorubicin may be due to the dissociation of destabilized nucleosomes from the broad epigenetic domains in cancer cells and normal cells of the heart, resulting in altered gene expression. In this context, anthracyclines like doxorubicin could be considered as epigenetic drugs that could severely disrupt the structure and expression of genes with the broad epigenetic domain.

## Broad epigenetic domains and cancer

Through integrative analysis of 1,134 genome-wide epigenetic profiles, mutations from > 8200 tumor-normal pairs and experimental data from clinical samples, Chen and colleagues discovered that tumor suppressor genes in normal cells were characterized by broad epigenetic domains. Broad epigenetic domains correlated with increased transcription elongation and enhancer activity, thus with extremely high gene expression. Moreover, a comparison of ENCODE H3K4me3 ChIP-seq data sets from 105 normal and 63 cancer samples showed that many tumor suppressor genes in cancer cell lines were associated with a shortening of their H3K4me3 domain [9].

More recently, Gopi and colleagues reported an integrative analysis of the epigenomes of 60 human cancer cell lines in the NCI-60 panel [75]. The oncogene *MYC* had a broad epigenetic domain in all 60 cancer cell types. GO annotation of genes with the broad epigenetic domain revealed cancer type-specific GO terms. The authors concluded that the identification of genes with the broad epigenetic domain in the various cancer cell types provided insights into the cancer phenotype. For example, in melanoma cells, the broad epigenetic domains had enrichment in the pigmentation GO terms. Further the broad epigenetic cancer-type specific patterns were distinct from those in normal cells.

In another recent study from the research groups of Vahid Asnafi and Salvatore Spicuglia, genes with the broad H3K4me3 domain in normal thymocytes and T acute lymphoblastic leukemia (T-ALL) were identified [76]. The authors applied an approach that differed from previous approaches [9, 42] to identify broad H3K4me3 domains; two cut-offs (high and low inflection points of ranked H3K4me3 peaks) identified broad H3K4me3 domains but also intermediates and sharp peaks [76]. As was the case with other cell types, the thymocyte genes with broad H3K4me3 domains were involved in tissue-specific functions. Further, the genes with the broad H3K4me3 domain were (1) highly dynamic throughout T cell differentiation, and (2) coded for transcriptional regulators involved in T-ALL leukemogenesis. The broad H3K4me3 domains were associated with H3K79me2, less so with H3K36me3, and displayed reduced RNAPII pausing. In analyses of nine primary T-ALLs and seven T-ALL-derived cell lines, the authors found that T-ALL oncogenes, including oncogenic long non-coding RNAs, had the broad H3K4me3 domain. Thus, T-ALL deregulated driver oncogenes had gained the broad H3K4me3 domain signature. The authors noted that there was a switch in the genes with the broad H3K4me3 domain when comparing normal T-cell precursors with leukemic cells; the T-ALL changed T-cell identity genes with cancer-related genes.

In inhibitor studies, the authors reported that the genes with the broad H3K4me3 domain were sensitive to elongation inhibitors (THZ1 (cyclin kinase CDK7 inhibitor), EPZ-5676 (DOTL1 inhibitor)) [76]. Of potential clinical significance, PBIT [2-(4-methylphenyl)-1,2-benzisothiazol-360 3(2H)-one], an inhibitor of the KDM5 family, strongly inhibited the proliferation of cell lines (e.g., Loucy) that had large numbers of broad H3K4me3 domains.

A comparison of the genes with the broad epigenetic (H3K4me3) domain in MCF7 [estrogen receptor (ER) + , luminal A], MDA-MB-231 (triple negative breast cancer cells), and MCF10A (basal epithelial cells representing a normal-like subtype) revealed the genes with this signature that were unique to each cell line [77] (manuscript in preparation). Analyses of these unique genes using Gene ontology and functional and pathway enrichment analysis using Genecodis and Ingenuity Pathway Analysis demonstrated that many genes containing the broad H3K4me3 domain code for sequence-specific DNA binding proteins, chromatin binding proteins, and proteins involved in the regulation of transcription within molecular functions. Hence, the broad epigenetic domain may mark key transcriptional regulators that drive a specific form of cancer.

In MDA-MB-231 breast cancer cells, which represent a more aggressive form of breast cancer, the *PPP1R15A* and *SPRY4* (Sprouty4) genes are marked by a broad epigenetic (H3K4me3) domain. The broad H3K4me3 domain is absent or considerably shorter in MCF7 and in “normal” MCF10A1 cells. The *PPP1R15A* gene encodes protein phosphatase 1 regulatory subunit 15A (a.k.a. *GADD34*, growth arrest and DNA damage-inducible gene 34). The PPP1R15A protein is a pleiotropic regulator that has key functions in multiple cellular processes including DNA damage pathways, endoplasmic stress responses, and chromatin remodeling. The latter is inferred from the observation that PPP1R15A binds to hSNF5/INI1, a component of the SWI/SNF chromatin remodeling complex [78]. Consistent with the general clinical relevance of perturbations in broad epigenetic domains, the *PPP1R15A* gene constitutes one of the genes in the hypoxia-related prognostic signature for breast cancer [79]. The biological importance of these findings is not restricted to breast cancer cells, because the *PPP1R15A* gene also has a broad H3K4me3 domain signature in multiple cell types, including erythroleukemic K562, and lung epithelial A549 cells [80]. The *SPRY4* is a tumor suppressor gene that inhibits the receptor-transduced mitogen-activated protein kinase signaling pathway and is a negative regulator of interferon signaling [81]. Silencing *SPRY4* expression in MDA-MB-231 cells increases cell proliferation, migration, and metastatic properties [82, 83].

In MCF7 cells, but not in MDA-MB-231 and MCF10A1 cells, the *FOXA1* gene has the broad epigenetic (H3K4me3) domain. Further, the *GATA3* gene has a broad epigenetic (H3K4me3) domain in MCF7 cells (Fig. 2B) but not in MDA-MB-231 cells, while in MCF10A1, the broad H3K4me3 domain of the *GATA3* gene is considerably shorter. FOXA1 and GATA3 have critical roles in ER + breast cancer in that these transcription factors form a regulatory network with ERα. These two transcription factors act as pioneer transcription factors which open condensed chromatin and are critical to maintain epithelial cell identity [84]. Reduced expression of *FOXA1* or *GATA3* results in increased metastatic progression and poor disease-free survival [54]. Highlighting these genes differentially marked with the broad H3K4me3 domain in MDA-MB-231 cells (*SPRY4*) and in MCF7 cells (*FOXA1* or *GATA3*) demonstrates the importance of these genes in maintaining the cellular properties of these two different types of breast cancer cells. Likewise, reducing expression of these genes may increase the metastatic potential of tumor cells and potentially lead to a poor prognosis. Reduction of *SPRY4* expression, for example, increases cancer stem cell properties in MDA-MB-231 cells, while reduced expression of *FOXA1* and *GATA3* by inhibiting the mitogen-and stress-activated protein kinase (MSK1) enhances the metastatic progression of ER + breast cancer. Silencing these genes with the broad H3K4me3 domain results in a more aggressive form of cancer.

## Broad epigenetic domains and brain function

The H3K4me3 chromatin distribution of sorted neuronal and non-neuronal nuclei in human post-mortem, non-human primate, and mouse prefrontal cortex were analyzed through extensive bioinformatics approaches of epigenomic and transcriptomic data, in the context of cell-type-specific regulation [44]. In mouse prefrontal cortex neurons, the broadest epigenetic (H3K4me3) domains (≥ 10 kb) were related to synaptic function and GABAergic signaling. Moreover, about 120 of the broadest epigenetic domains, annotated to human genes largely involved in dopaminergic and glutamatergic signaling, were conserved in chimpanzee, macaque, and mouse cortical neurons. In agreement with previous results, position of cell type-specific broad epigenetic domains correlated with the expression of genes that control cell identity. From a clinical perspective, this study supports the role of genes with the broad epigenetic domain in psychiatric disorders such as autism and schizophrenia [44].

Behavioral studies in mice (i.e., experiments on contextual fear conditioning) established that broad epigenetic domains are specifically present in genes activated during memory formation in cornu ammonis (CA1) neurons of the hippocampus (i.e., *Calm1*, *Fos*, and *Npas4*). Because *Fos* and *Npas4* are immediate early genes, it appears that genes marked by a broad epigenetic domain are poised for activation and become highly expressed following stimulus exposure [85].

## Breadth of the broad epigenetic domain as a key element in three-dimensional chromatin interactions

**Fig. 5:**
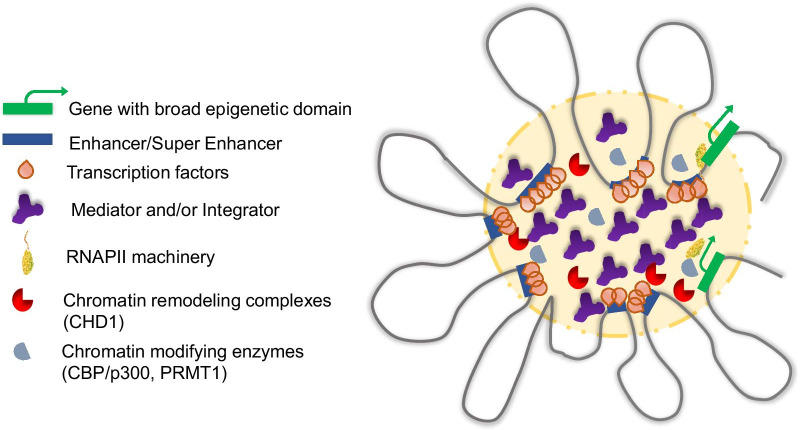
Transcription factory containing enhancer, super-enhancer, and broad epigenetic domain interactome. The transcription factory, a phase-separated condensate, contains high concentrations of chromatin modifying enzymes (e.g., CBP/p300, PRMT1), chromatin remodelers, Mediator (and/or Insulator), topoisomerases, and cell-type specific transcription factors. The transcription factors contribute to the localization of the enhancer/super-enhancers and interaction with the genes with the broad epigenetic domain

It has been shown that compared to H3K4me3 narrow enrichment tracts, broad epigenetic (H3K4me3) domains were preferentially involved in 3D chromatin interactions that were required for transcription of the relevant genes [10, 61, 86, 87] (Fig. 5). These studies used RNA polymerase II-associated ChIA-PET (chromatin interaction analysis by paired-end tag sequencing) data in K562 chronic myelogenous leukemia cells and MCF-7 breast cancer cells [86], ChIA-PET, Hi-C (a method based on chromosome conformation capture), and Hi-ChIP (HiC chromatin immunoprecipitation) data in K562, MCF7 and GM12878 lymphoblastoid cells [87], OCEAN-C (Open Chromatin Enrichment And Network Hi-C) in U266 myeloma cells, RPMI-8226 multiple myeloma cells and GM12878 [61] and 3C (chromosome conformation capture) in tissue from the cerebellum of mice [10]. They demonstrate the relevance of broad epigenetic domains, particularly in the context of cancer. OCEAN-C, combining Hi-C and FAIRE-seq, detects hubs of open chromatin interactions (HOCIs) [61]. It involves a phenol–chloroform extraction and isolation of the DNA to be sequenced from the aqueous phase, thus resulting in the detection of nucleosome-depleted-region or open-chromatin interactions. This method would be more specific than the other methods above as it relies on enhancers being nucleosome-depleted regions. It was evidenced that OCEAN-C is a method able to identify interactions between open chromatin regions which are essential for gene transcription. Moreover, pathway enrichment analysis of the genes with a broad epigenetic domain overlapping with HOCIs, revealed that the majority of top enriched pathways (transcriptional misregulation in cancer, thyroid hormone synthesis, p53 signaling pathway, cell cycle, microRNAs in cancer) were related to cancer in U266 cells [61]. Alterations in the p53 signaling pathway, which is involved in regulating cell cycle, cell death (apoptosis), and chromatin repair, are often altered in cancer cells. Further alterations in microRNAs can change the mRNAs that are translated in cancer cells.

Super-enhancers have been identified by the presence of the Mediator complex (and/or Integrator complex), H3K27ac, and CBP/p300 [88–92]. Note that Mediator and Integrator are major multiprotein complexes that associate with RNA polymerase II. Although Mediator and Integrator play important roles in enhancer/super-enhancer/promoter interactions, these complexes have enhancer/super-enhancer specific functions (e.g., Integrator requirement for immediate-early gene induction and expression) [91, 92]. Transcription factor circuits, such as the TCF4 circuit in mouse neural stem cells, appear to be important in the formation of the 3D interactions between super-enhancers and genes with the broad epigenetic domain. Key in forming these interactions is the Mediator complex which binds to transcription factors, chromatin remodelers (SWI/SNF), chromatin modifying complexes (KMT2, CBP/p300, NuRD histone deacetylase complex), coactivators, cohesin and other proteins. One study reported the detection of genes, enriched in transcriptional regulators, with superposed super-enhancer and broad epigenetic domain on the basis of the broad H3K4me3 domain tracking with Mediator, transcription factors (TCF4, NF1), and chromatin remodelers (CHD7) [89]. However, whether the transcription factors are binding to the broad epigenetic domain directly or indirectly could be questioned. The transcription factor could be binding to an enhancer or super-enhancer and cross-linked to the broad epigenetic domain when interaction between the regulatory region and broad epigenetic domain occurs.

The interactions between the enhancers/super-enhancers and broad epigenetic domain marked genes are dynamic. Contributing to this dynamic interaction may be polyADP-ribosylation of the histones [93, 94]. During transcription initiation and elongation, topoisomerases I and II are involved in the release of torsional stress generated during transcription [95, 96]. The action of these enzymes in generating single-stranded and double-stranded DNA breaks could result in the recruitment of polyADP polymerase. Of the histone modifications, polyADP ribosylation is the modification most rapidly added and removed.

## A model for the establishment of the broad epigenetic domain

It remains to be determined how broad epigenetic domains are established for specific genes. However, transcription factor circuits that include pioneer and cell type specific transcription factors binding to enhancers, super-enhancers and upstream promoter elements would likely establish the interactome between these regulatory elements (e.g., KLF5 in human epithelial cancers; TCF4 in neural stem cells) [89, 97, 98]. The transcription factors binding to and mediating these 3D interactions may be assembled into transcription factories [99–101], which may have the properties of a liquid–liquid phase-separated condensate [102] (Fig. 5). Transcription factors also recruit chromatin modifying enzymes [protein arginine methyltransferase 1 (PRMT1), CBP/300] to enhancers/super-enhancers and promoters [51, 60, 103–105]. PRMT1 is required to establish the active chromatin state and is needed for hematopoietic differentiation in avian and mammalian cells [103, 105, 106]. Interaction between the locus control region (a super-enhancer) and the β-globin promoter in the β-globin gene (broad epigenetic domain) is prevented when PRMT1 was knocked down. The interaction of the locus control region with the globin promoter increases transcriptional bursting (burst fraction and size) [60].

PRMT1 binds to CBP/p300 as well as many other proteins. The PRMT1 product, H4R3me2a, stimulates that activity of CBP/p300 to acetylate H3K27. Further, CBP/p300 co-condenses with transcription factors, and the co-condensation modulates transcriptional bursting. Thus, PRMT1 may have a role in activation of enhancers via the enzyme’s recruitment of CBP/p300 and H3K27 acetylation and in interaction of the enhancer/super-enhancer/locus control region with the target promoter, a step that can take place before enhancer activation [105]. Recently, it was reported that PRMT1 regulates the gene expression program in mouse mature β-cell and is required for maintaining mature β-cell identity [51]. These observations suggest that PRMT1 plays a pivotal role in the establishment of the broad epigenetic domain interactome.

The recruitment of the general transcription machinery would lead to the first round of transcription, which is often needed to recruit chromatin modifiers (KATs) and chromatin remodelers (CHD1 [63],), and would establish histone PTMs (e.g., acetylated histones, ubiquitinated H2B) [29, 107]. The assembly of chromatin modifiers, chromatin remodelers, coactivators, Mediator, long non-coding RNA, and other regulatory molecules would contribute to the highly modified, chromatin dynamic state of genes with the broad epigenetic domain. Also for consideration is the interaction of the enhancer/super-enhancers with the target gene in establishing the broad epigenetic domain. KMT2B (MLL4) at super-enhancers is involved in the genesis of the broad epigenetic domain [10]. However, the enzyme’s role in creating chromatin accessibility at enhancers/super-enhancers does not require the enzyme’s catalytic activity, which produces H3K4me1 [108, 109]. A model is emerging in which interactions (multivalent/hydrophobic) between the intrinsic disordered domains of Mediator (e.g., MED1 subunit), CBP/p300, and transcription factors form phase-separated condensates [60, 102, 110] (Fig. 5). Integrator is also likely involved in the formation of condensates, a role that future studies will need to address. The condensates would support long residence times of the transcription factors and chromatin modifying/remodeling factors on the enhancers/super-enhancers/locus control regions/promoters resulting in increased transcriptional burst frequencies and transcriptional consistency [90]. As discussed by Zamudio et al. [102], the phase-separated three-dimensional condensate model, and within this model the chromatin organization and expression of genes with broad epigenetic domain would help in our understanding of pathological events leading to disease.

## Limitations with analyses of broad epigenetic domains

The bioinformatic pipeline for peak detection from ChIP-seq data often includes a step where the issue of duplicate reads poses the challenge whether these reads should be filtered out or kept in. The duplicates are often artifacts from PCR bias but sometimes can be due to genomic regions that inherently get overamplified resulting in artifacts and noise [111]. These regions, termed blacklisted regions, often have underlying sequences enriched with repeat sequences and elements. Removal of these regions often improve sequence quality leading to better signal-to-noise ratio [112]. Unfortunately, they are often found in open chromatin regions and removing them may result in miscalling of pileups, underestimation of signal level and adversely affect downstream integrative and differential analysis leading to wrong biological interpretation [113, 114]. Each peak calling tool addresses this filtering issue differently by setting threshold cut offs and parameters that only “true peaks” can satisfy. For MACS2, a smaller value for lower cut off allows detection of larger number of regions, increasing the chance of confounding factors to affect the outcome of the model [115]. For tools utilizing the Hidden Markov Model, the strictness of peak identification is often based on the cut off posed on the posterior probability [116]. The latter method is considered better for peak determination as it provides a measure of confidence compared to simple binary outputs [117]. Unlike narrow peaks, broad domains are typically of low intensity which results in poor model training and technical variabilities [118].

Of the various broad peak calling tools, MACS2 (broad setting) is the most used. The other tools include: Broadpeak, Zinba, RSeg, SICER, and histoneHMM [115, 119–123]. Most of these tools work by filtering duplicates and aggregating nearby peaks to determine broad peaks. The algorithm specific region bias, probability threshold and parameters vary from tool to tool. The variation in stringency levels among the tools lead to variation in identified peaks raising the issue of defining optimum broad domain parameters. This issue of default operating resolution among the tools is not limited to bivariant setting but is also seen with tools for multivariant peak calling such as ChromHMM [124]. It is recommended not to feed broad peak data into the ChromHMM tool as it conflicts with the resolution at which it determines the combinatory states. Hence, characterization of broad domains requires careful consideration of peak calling applications and thresholding parameters. These factors are still being optimized.

A note of caution is warranted regarding the interpretation of these data when it comes to enhancers and super-enhancers. In a previous study, H3K27ac was deemed the mark of choice to identify a large fraction of super-enhancers, while minimizing erroneous DNA regions. Thus, in many of the bioinformatic studies, super-enhancer prediction entailed stitching H3K27ac peaks together within 12.5 kb of one another in the linear genome [125]. However, we argue that this labeling of H3K27ac broad domains as enhancers or super-enhancers is prone to errors as it does not consider other marks such as H3K4me1 and does not meet the classical or functional definition of an enhancer or super-enhancer (see Glossary for definition of terms). In brief, calling a H3K27ac broad domain an enhancer or super-enhancer is a gross oversimplification that ignores the intricate interactions governing gene regulation. In fact, many histone PTMs mirrored the H3K4me3 mark either as sharp peaks near the TSSs or as broad peaks in the gene bodies, including H3K27ac [9, 86] (Fig. 2B), leading to terminology confusion and inconsistencies.

We envisage that each gene allele with a broad epigenetic domain is in a state of flux with CHD1-mediated nucleosome dissolution and reformation along the gene body (Fig. 4A). The nucleosomes would be highly modified. A limitation of the current picture we have at present is that the ChIP-seq and accessibility measurements are the average of these events in millions of the cells in the analyses. These assays can become even more complicated when using clinical specimens in which the intensity of the histone PTM levels (e.g., H3K27ac) may reflect phenotypic heterogeneity [126]. To further explore the dynamics of the broad epigenetic domain, single cell ChIP-seq and accessibility assays are required [127]. MAPit, which provides single allele accessibility information, would be an excellent method to view the heterogeneity of nucleosome and protein (transcription factor) occupancy of specific alleles with the broad epigenetic domain in the study population of cells. The MAPit method applies an exogenous DNA methyltransferase, Chlorella virus protein M.CviPI, which methylates GC when not occupied by a nucleosome or DNA-binding protein [128–130]. In addition to visualizing chromatin accessibility for each allele in the population, the method also shows the DNA methylation (five methyl CG) for each allele. A similar strategy but using a different methyltransferase (DNA N^6^-adenine methyltransferase) is called Fiber-seq [131]. For both methods (MAPit and Fiber-seq) long read sequencing is required (single-molecule circular consensus sequencing with a Pacific Biosciences instrument).

## Concluding remarks

Genes with the broad epigenetic signature have critical roles in cell identity and essential cell functions. Thus, identification of marked genes provides information about essential genes in normal and diseased states of cells as highlighted in our example of genes with the broad epigenetic domain in human breast cancer cells and in chicken polychromatic erythrocytes. Of clinical relevance, epigenetic therapy approaches [76, 132, 133] that alter the expression of genes with the broad epigenetic domain must be cognizant that silencing these genes may make the cancer more aggressive as shown with *SPRY4* in triple negative breast cancer. Mutations in the DNA sequence transcription factor binding sites in enhancers and super-enhancers, altered expression of transcription factors, and expression of oncohistones (replacement histones such as H3.3) will impact the formation and function of the broad epigenetic domain marked genes [101]. The chromatin structure of the broad epigenetic domain is very dynamic, with histone PTMs, nucleosome dissolution/ reassembly, and histone variant exchange. The enzymes catalyzing these events alter the function of each other by interacting and mutually modifying each other. Layered on these events is metabolism which provides the factors fueling the enzymes [3] (Fig. 1). The transcription factories housing the broad epigenetic domain and its interactome would have a high demand for energetic metabolites such as ATP, S-adenosyl methionine, acetyl-CoA, NAD, and α-ketoglutarate. Disturbance in the availability of these metabolites in the diet or as a consequence of disease may impact the expression of genes with the broad epigenetic domain [134, 135]. Exploring the orchestration of the metabolites and epigenetic players in the formation and maintenance of transcription factories/hubs with genes with a broad epigenetic domain and their interactome of enhancers and super-enhancers is a prerequisite to understanding cell differentiation and disease.

## Data Availability

Unprocessed raw files are available from the corresponding author on reasonable request.

## Appendix: Glossary

- **Broad H3K4me3 (or H3K27ac) domain**: H3K4me3 (or H3K27ac) ChIP-seq peaks which, when ranked by size, constitute the top 5% broadest H3K4me3 (or H3K27ac) domains. Each peak is attributed to the gene with the closest transcription start site [42]. It should be noted that broad domains identified in the literature as broad H3K4me3 domains are also characterized by the widely spread presence of many other marks [9, 47]. However, this broad distribution of other marks has been overlooked in the literature.
- **Cell identity genes:** Development regulator genes that are specific to each cell type. Their expression often serves as crucial biomarker for diseased and healthy cells.
- **Enhancer**: “Enhancers are classically defined as cis-acting DNA sequences that can increase the transcription of genes. They generally function independently of orientation and at various distances from their target promoter(s)” [137]. An active enhancer binds multiple transcription factors, which recruit the Mediator complex, facilitating the interaction of the loaded enhancer to the cognate promoter via the RNA polymerase II and the transcription machinery. This process is called looping [138]. As enhancers can be located tens to hundreds of kb away from the gene(s) they regulate, the identification of enhancers and their target gene(s) can be challenging. Features generally exploited to identify putative enhancers are DNase I hypersensitivity, H3K4me1 and H3K27ac marks as well as association of CBP/p300, which acetylates H3K27 among other targets [138]. It must be noted that few of the predicted super-enhancers have been functionally investigated by deletion [139].
- **Super-enhancer**: Cluster of enhancers [88, 138]. It is generally believed that “typical” enhancers are involved in the transcription of housekeeping genes, while super-enhancers only regulate cell-type-specific genes that confer cell identity [138]. However, it has been argued that an isolated enhancer can achieve a robust transcriptional output, while a super-enhancer acts in a partially redundant manner to fine tune the transcriptional output of its cognate gene(s) [139].

## Abbreviations

CHD1: Chromodomain helicase DNA binding protein 1
ChIP-seq: Chromatin immunoprecipitation sequencing
ER: Estrogen receptor
FACT: Facilitates Chromatin Transcription
FAIRE: Formaldehyde-Assisted Isolation of Regulatory Elements
H3K4me3: Histone H3 trimethylated at lysine 4
HDACs: Histone deacetylases
KATs: Lysine acetyltransferases
PAF: Polymerase-Associated Factor
PRMT: Protein arginine methyltransferase
PTMs: Post-translational modifications
T-ALL: T acute lymphoblastic leukemia
TSSs: Transcription start sites
